# Pilot application of a sustainable and disaster-resilient infrastructure assessment framework: Evidence from Bandung, Indonesia

**DOI:** 10.4102/jamba.v18i1.2034

**Published:** 2026-06-02

**Authors:** Aden Firdaus, Krishna S. Pribadi, Muhamad Abduh, Saut A. Sagala

**Affiliations:** 1 Department of Civil Engineering, Faculty of Civil and Environmental Engineering, Bandung Institute of Technology, Bandung, Indonesia; 2 School of Regional and Rural Planning, Bandung Institute of Technology, Bandung, Indonesia

**Keywords:** sustainable infrastructure, disaster resilience, urban planning, infrastructure assessment, risk and vulnerability, recovery, resilience indicators, Indonesia

## Abstract

Urban infrastructure in developing countries faces mounting pressures to achieve both sustainability and disaster resilience amid limited data and institutional capacity. This study presents the first empirical application of a Sustainable and Disaster-Resilient Infrastructure (SDRI) assessment framework to the road and bridge network of Bandung, Indonesia. Using secondary data from the Bandung City Government, the National Disaster Management Agency, and the Central Bureau of Statistics, complemented by expert interpretation, the framework evaluates seven dimensions – risk, vulnerability, resistance, recovery time, social, economic and environmental. The assessment reveals a highly unbalanced profile: while Bandung demonstrates strong reactive capacities in the recovery time (3.29) and social (3.62) dimensions, its proactive components, such as risk (0.00) and vulnerability (0.43), remain critically weak. The city’s infrastructure thus exhibits resilience in restoring function after disruption but lacks anticipatory strength to mitigate risks beforehand. Economic fragility (1.50) and the absence of integrated hazard-based planning further constrain systemic resilience. Methodologically, the study validates the SDRI framework’s feasibility for data-limited contexts and its adaptability across infrastructure types. The findings underscore the need to transition from reactive repair-based practices towards proactive, risk-informed, and adaptively governed infrastructure planning.

**Contribution:**

By operationalising the sustainability–resilience nexus within an actual municipal context, this research bridges the gap between conceptual frameworks and practical assessment, offering a replicable approach for urban policymakers seeking to institutionalise resilience in sustainable infrastructure development.

## Introduction

Infrastructure systems in developing countries are increasingly challenged by the dual imperatives of sustainability and disaster resilience. Indonesia, located in the Pacific Ring of Fire, is among the world’s most disaster-prone nations, experiencing more than 17 000 natural hazard events between 2016 and 2020 (National Disaster Management Agency [BNPB] 2021). These recurrent hazards – earthquakes, floods, volcanic eruptions and landslides – pose significant risks to infrastructure networks that are critical for national and local development. The integration of disaster resilience within sustainable infrastructure planning has therefore become an essential priority for achieving national development objectives and the Sustainable Development Goals (SDGs), particularly SDG 9 on resilient infrastructure and SDG 11 on sustainable cities.

Over the past decade, the Indonesian government has introduced several regulatory instruments to mainstream sustainability and resilience principles into infrastructure development. Key frameworks include Presidential Regulation No. 87 of 2020 on the *National Disaster Management Master Plan (2020–2044)*, Presidential Regulation No. 111 of 2022 on *SDG Implementation* and Ministerial Regulation of Public Works and Housing (No. 9 of 2021) on *Sustainable Construction Guidelines*. These regulations collectively aim to institutionalise Sustainable and Disaster-Resilient Infrastructure (SDRI) practices across planning, construction and operational phases. However, despite these advances, implementation remains fragmented, constrained by limited data integration, inconsistent assessment mechanisms and weak inter-agency coordination (Husnita & Ghaniyyu 2020; Trigunarsyah 2021; Vallery 2020).

The concept of SDRI combines the long-term sustainability of infrastructure systems – covering environmental, social and economic dimensions – with their capacity to withstand, adapt to, and recover from disruptions (Gillespie-Marthaler et al. 2019; Nelson et al. 2020). Sustainability without resiliency is vulnerable to shocks, while resiliency without sustainability can lead to resource inefficiency and social inequity (Chhibber & Laajaj 2007). The SDRI framework developed in previous research (Firdaus et al. 2025) integrates these dual perspectives through seven key dimensions: economic, environmental, social, risk, resistance, vulnerability, and recovery speed. While the framework has been conceptually validated through expert judgement and statistical analysis, its empirical applicability has not yet been tested in real-world contexts.

This study addresses that gap by applying the SDRI assessment framework to evaluate the sustainability and resilience performance of road and bridge infrastructure in Bandung City, Indonesia. Bandung, a major urban centre characterised by rapid urbanisation, complex infrastructure systems and exposure to multiple hazards – including the active Lembang Fault – represents an ideal testbed for piloting the framework. Using available secondary data complemented by expert interpretation, this research provides an operational demonstration of the framework’s capacity to assess infrastructure conditions, identify strengths and weaknesses across the seven dimensions, and visualise the city’s sustainability – resiliency profile.

The contribution of this paper is threefold. Firstly, it presents an empirical application of the SDRI framework, testing its feasibility using actual municipal data. Secondly, it generates diagnostic insights into Bandung’s infrastructure sustainability and disaster resilience performance, identifying systemic gaps and priority areas for improvement. Thirdly, it advances methodological understanding by demonstrating how a multi-dimensional framework can be operationalised in developing-country contexts with limited data. The findings are expected to inform policymakers, practitioners and researchers on the practicality of applying integrated sustainability–resilience assessments to guide infrastructure planning and management.

### Theoretical background

#### Sustainability and resiliency in infrastructure systems

Infrastructure constitutes the physical foundation of socio-economic development, supporting essential services such as mobility, logistics and connectivity (United Nations Environment Programme 2021). The concept of sustainability in infrastructure emphasises the capacity of assets and systems to deliver economic efficiency, social equity and environmental stewardship over their life cycle (Gibson 2006; World Commission on Environment and Development (WCED) 1987). A sustainable infrastructure system, therefore, seeks to minimise negative externalities – such as emissions, resource depletion or social displacement – while maximising long-term value creation.

In parallel, disaster resilience refers to the ability of a system to resist, absorb, adapt to, and recover from hazard-induced disruptions while maintaining essential functions (Holling 1973; United Nations Development Programme [UNDP] 2015). For infrastructure, resilience encompasses robustness of design, redundancy of critical functions, resourcefulness in crisis response, and rapidity in recovery (Bruneau et al. 2003). The two paradigms are interdependent: sustainable systems without resilience remain vulnerable to shocks, whereas resilient systems lacking sustainability may perpetuate inefficiencies or inequities (Ahern 2011; Chhibber & Laajaj 2007).

Integrating these paradigms requires attention to seven broad dimensions commonly recognised in the literature – economic, environmental, social, risk, resistance, vulnerability and recovery speed (Gillespie-Marthaler et al. 2019; Nelson et al. 2020). Economic sustainability relates to efficiency and affordability; environmental sustainability to ecological preservation and climate compatibility; social sustainability to equity and inclusiveness; risk to the type of disaster and hazard; resistance to performance degradation and capacity; vulnerability to sensitivity and individual risk; and recovery speed. Collectively, these dimensions provide a holistic lens for evaluating infrastructure performance across both sustainability and resilience domains.

#### Limitations of existing assessment approaches

A large body of work has sought to assess the sustainability or resilience of infrastructure, yet integration remains limited. Traditional sustainability assessments – such as Leadership in Energy and Environmental Design – Neighborhood Development (LEED-ND), Envision or Building Research Establishment Environmental Assessment Method (BREEAM) – emphasise environmental and resource efficiency but inadequately capture adaptive capacity and post-disaster recovery (Bocchini et al. 2014). Conversely, resilience-focused indices, including the Disaster Resilience Scorecard (Khazai, Anhorn & Burton 2018) and the Infrastructure Resilience Index (Shrestha & Kawasaki 2020), prioritise hazard preparedness and system robustness while often neglecting socio-economic and environmental sustainability considerations.

Moreover, most frameworks are developed for high-income contexts, requiring detailed data and institutional maturity rarely available in developing countries (Trigunarsyah 2021). In Indonesia, despite policy commitments to SDRI, assessment practices remain fragmented across sectors – environmental impact analysis (Analisis Mengenai Dampak Lingkungan [AMDAL] [*Environmental Impact Assessment*]), technical feasibility studies and post-disaster evaluations operate independently with little integration (Husnita & Ghaniyyu 2020). The absence of a unified assessment mechanism limits policymakers’ ability to benchmark progress or prioritise interventions. Therefore, there is a pressing need for a framework that is integrative, data-efficient and context-adaptable for developing-country urban systems.

#### Overview of the sustainable and disaster-resilient infrastructure framework

**FIGURE 1:**
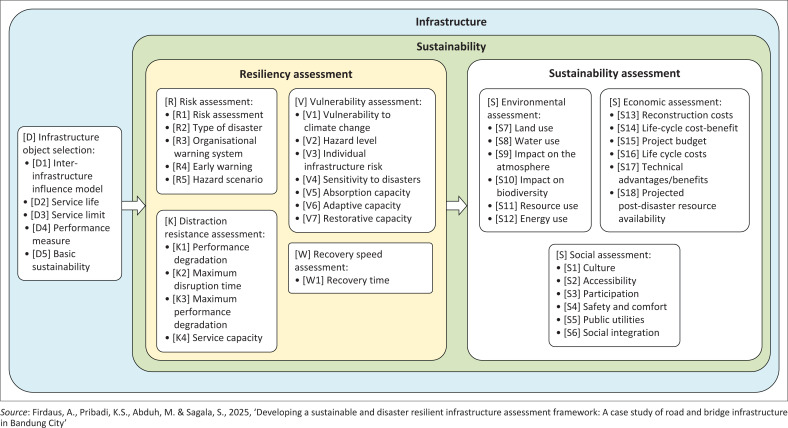
Sustainable and disaster-resilient infrastructure assessment framework.

To address these gaps, an SDRI assessment framework has been developed, later refined and statistically validated in subsequent research (Firdaus et al. 2025) (Figure 1). The framework conceptualises infrastructure performance through seven interrelated dimensions – economic, environmental, social, risk, resistance, vulnerability and recovery speed – each represented by measurable indicators derived from literature, international standards and Indonesian regulatory instruments (e.g. Perpres 87/2020; Permen Pekerjaan Umum dan Perumahan Rakyat [PUPR] [*Ministry of Public Works and Housing*] 9/2021).

Each dimension contains multiple criteria and indicators designed to evaluate both sustainability and resilience attributes:

- *Economic* indicators include cost efficiency, resource utilisation and long-term value.
- *Environmental* indicators assess energy efficiency, emission reduction and ecosystem protection.
- *Social* indicators measure inclusiveness, accessibility and community benefit.
- *Risk* indicators evaluate disaster risk assessment and its use, type of disaster, organisational ability to process risk data to provide warnings, early warning, hazard scenario.
- *Resistance* indicators examine the decrease in infrastructure performance.
- *Vulnerability* indicators capture infrastructure vulnerability, hazard level (exposure), risk of an infrastructure element, infrastructure sensitivity, infrastructure absorption capacity, adaptive capacity and restorative capacity.
- *Recovery Speed* indicator assesses the recovery time.

The framework operates as a multi-criteria assessment tool, enabling quantitative scoring and qualitative interpretation across these seven dimensions. It has been internally validated using expert surveys and statistical reliability tests, confirming its conceptual soundness and internal consistency (Cronbach α = 0.803). However, prior to this study, the framework had not been applied empirically to a real case. The present research, therefore, serves as the first pilot application, testing its feasibility for evaluating urban infrastructure systems – specifically the road and bridge network of Bandung City – using existing data sources and expert interpretation.

## Research methods and design

### Case study: Ahmad Yani corridor of Bandung City

Bandung City, the capital of West Java Province, is one of Indonesia’s major urban centres with a population exceeding 2.5 million (Statistik 2022). Geographically located in a basin surrounded by volcanic highlands, the city is exposed to multiple hazards, including earthquakes, floods and landslides. The Lembang Fault, situated approximately 10 km north of the city centre, poses a significant seismic risk, while seasonal hydrometeorological hazards – exacerbated by rapid urbanisation and inadequate drainage – cause recurrent flooding in low-lying areas (BNPB 2021).

Bandung’s infrastructure network plays a critical role in regional connectivity and economic activity. Its road and bridge system links the Bandung Basin to the Greater Jakarta region and serves as the primary corridor for goods and commuter mobility. However, the city faces structural and operational challenges, such as limited road capacity, ageing bridges and high maintenance demands (Bandung 2020). At the same time, urban expansion and changing climate patterns increase infrastructure exposure and vulnerability to disasters.

The Ahmad Yani corridor was selected as the focal case within Bandung’s road and bridge infrastructure system due to its strategic, representative and data-accessible characteristics. As one of the city’s primary arterial routes, Jalan Ahmad Yani connects the eastern and central districts of Bandung, serving as a critical economic and mobility axis linking industrial, commercial, and residential zones. The corridor is characterised by high traffic volume, mixed land use, and exposure to multiple hazards, including recurrent flooding near the Cicadas and Antapani areas and potential ground shaking from the nearby Lembang Fault. These conditions make it a relevant microcosm of Bandung’s broader infrastructure challenges – balancing economic functionality with sustainability and disaster resilience requirements. Moreover, the corridor has been the subject of several municipal and national infrastructure programmes, providing relatively comprehensive data availability from agencies such as the Water Resources and Road Agency (Dinas Sumber Daya Air dan Bina Marga [DSDABM] [*Department of Water Resources and Road/Highway*]). Its selection, therefore, enables a contextually rich yet analytically manageable pilot assessment to test the operational applicability of the SDRI framework.

**FIGURE 2:**
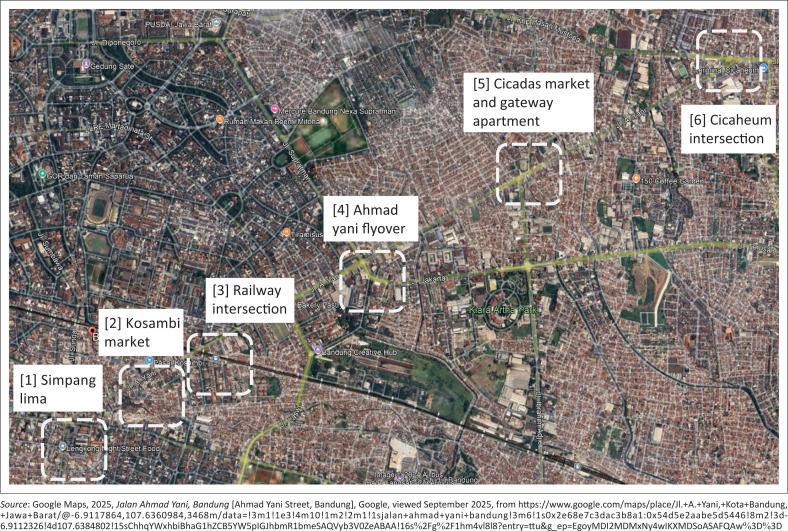
Ahmad Yani corridor, Bandung.

The empirical assessment focused on the Ahmad Yani corridor, one of Bandung City’s most critical urban arteries, connecting the city centre to its eastern districts. This corridor encompasses six representative locations that capture the diversity of urban functions and resilience challenges along the route. Figure 2 illustrates the spatial distribution of the study locations in Bandung City. The numbered locations (1–6) represent key infrastructure segments assessed in this study. The Simpang Lima (1) intersection serves as a pivotal node of urban mobility, characterised by high traffic volume and strategic importance in regional connectivity. The Kosambi Market (2) area represents a dense economic hub that anchors local commerce and daily trade activities. The railway intersection (3) marks a critical multimodal junction facilitating both passenger and freight movement, underscoring the corridor’s logistical relevance. The Ahmad Yani Flyover (4), an elevated structure traversing the corridor, was designed to mitigate congestion and maintain transport continuity during peak periods and hazard events. Further east, the Cicadas Market and Gateway Apartment zone (5) reflects a mixed-use urban typology that integrates residential and commercial functions, highlighting socio-economic interdependencies within the corridor. Finally, the Cicaheum intersection (6), located adjacent to the city’s primary bus terminal, functions as a vital gateway for public transportation and intercity connectivity. Together, these six segments form a representative cross-section of Bandung’s urban infrastructure system, linking the technical, social and economic dimensions that are central to evaluating urban resilience and sustainability.

### Assessment framework and data

The assessment employed the SDRI framework, previously developed and validated through expert evaluation and statistical testing (Firdaus et al. 2025). The framework operationalises seven interrelated dimensions – economic, environmental, social, risk, resistance, vulnerability and recovery speed – each represented by a set of measurable indicators. These indicators are derived from international literature, for example, Gillespie-Marthaler et al. (2019) and Nelson et al. (2020), national regulations (Perpres 87/2020; Permen PUPR 9/2021), and local planning documents (Rencana Pembangungan Jangka Menengah Daerah [RPJMD] [*Regional Medium-Term Development Plan*] Bandung 2018–2023).

The SDRI assessment framework is conceptually derived from Nelson et al. (2020), a model that has advanced the understanding of resilience by framing it as a dynamic balance between system sustainability and adaptability within complex socio-technical networks. It emphasised feedback processes and interdependencies across physical, social, and institutional components. Building on this foundation, the SDRI framework operationalises these principles for the infrastructure sector, translating abstract system attributes into seven quantifiable dimensions: *risk, vulnerability, resistance, recovery time, social, economic*, and *environmental*. Whereas Nelson’s framework was primarily conceptual, focusing on dynamic interactions, the SDRI model introduces a structured indicator-based assessment that allows empirical measurement and comparison across assets and local contexts. This refinement enhances practical applicability by bridging system theory and policy-oriented evaluation, enabling the systematic assessment of infrastructure resilience using available data while retaining its grounding in sustainable systems thinking.

In addition to using publicly available secondary data, this study also incorporated institutional inputs from the Bandung City Department of Highways, Irrigation and Bridges (Dinas Sumber Daya Air, Bina Marga, dan Bina Konstruksi – DSDABM). DSDABM is the municipal agency responsible for planning, constructing and maintaining Bandung’s road and bridge infrastructure, including coordination with provincial and national authorities for network integration and asset management. Several rounds of technical discussions were conducted with the DSDABM official between November 2024 and March 2025. These discussions clarified data gaps, validated infrastructure information and supported the contextualisation of indicator scoring. During these consultations, DSDABM provided supporting documents such as the Governor of West Java Decree No. 620/883/2022 on the Primary Road Network Classification by Function, the Governor of West Java Decree No. 620/884/2022 on Provincial Road Status, the official Bandung City Road Map, the Bloomberg Liveable City Report, pedestrian facility guidelines, inspection reports and inventories of cultural heritage buildings. Supplementary materials, including photographic documentation and administrative decrees related to provincial road management, were also reviewed. These official datasets and institutional insights contributed to improving the accuracy and contextual grounding of the SDRI assessment.

To maintain consistency with the validated framework, the same indicator structure and scoring logic were used. Each indicator was scored based on available evidence and cross-checked with regulatory targets or technical benchmarks. When quantitative data were incomplete, qualitative assessments were applied. These used ordinal scales (low-, medium-, high) to capture relative performance levels. This approach ensures that the framework can function in data-constrained environments typical of local governments in developing countries.

The focus of this exercise is feasibility testing, not stakeholder validation. Hence, the scoring and analysis constitute a *pilot author-based assessment* intended to evaluate whether the framework can be operationalised using existing data, identify challenges in data availability and reveal indicative sustainability–resilience patterns.

### Assessment process

The application of the SDRI framework in Bandung followed four analytical steps:

- Data compilation and indicator mapping: All relevant secondary data were compiled and organised according to the SDRI indicator matrix. Each dataset was then linked to its corresponding sustainability or resilience dimension. Where data were missing, values were inferred using supporting documents or expert interpretation.
- Parameter exception: Environmental parameters were not assessed because road and bridge infrastructure are considered passive in terms of resource utilisation. These parameters include water use, atmospheric impact, biodiversity and land effects, resource consumption and energy use. They are more relevant to infrastructure that actively consumes resources during operation. The exclusion of the environmental dimension from this assessment, while justified by the predominantly passive operational nature of road and bridge infrastructure, limits the SDRI model’s holistic completeness. Environmental interactions – particularly those related to construction material life cycles, land-use change and stormwater runoff – may indirectly influence long-term sustainability and resilience outcomes (Lee, An & Kim 2025). Although these aspects are not explicitly captured in the current framework, they remain important considerations.
- Indicator scoring: Each indicator was assigned a score between 0 and 5, where 0 represents inadequate performance and 5 indicates optimal achievement relative to the expected standard or target. Quantitative indicators (e.g. road density, maintenance ratio) were normalised, while qualitative indicators (e.g. community participation, institutional coordination) were scored using a structured judgement scale.
- Normalisation and aggregation: Scores were normalised and averaged within each sub-dimension to produce dimension-level indices. Equal weighting was applied across dimensions to maintain neutrality in this pilot phase. Further empirical calibration would require larger-scale stakeholder participation.
  The aggregated results generated two composite indices:
  ▪ A sustainability index (economic, environmental, social) and
  ▪ A resilience index (risk, resistance, vulnerability, recovery).
- Visualisation and interpretation: The resulting indices were visualised using radar (spider) diagrams to illustrate the performance profile of Bandung’s infrastructure across the assessed dimensions. The visualisation provides an intuitive overview of the city’s strengths, weaknesses and inter-dimensional imbalances. It also serves as a diagnostic tool for policy interpretation and planning improvement.

### Validity and reliability considerations

Although this pilot application relied on expert judgement, methodological rigour was maintained. This was achieved by ensuring consistency with the validated SDRI framework and applying transparent scoring criteria. The original framework’s internal reliability (Cronbach’s α = 0.803) supports confidence in the indicator design. Nevertheless, given the single-assessor approach and limited data availability, results are interpreted as indicative rather than conclusive. Future multi-stakeholder validation is recommended to strengthen robustness and reproducibility.

## Results

### Overall sustainable and disaster-resilient infrastructure profile of Bandung

The application of the SDRI framework produced a composite performance profile for Bandung’s road and bridge infrastructure (Table 1). The overall SDRI index indicates that the city’s infrastructure exhibits moderate sustainability and limited resilience performance, reflecting the transitional state of infrastructure development in an urban area experiencing both modernisation and persistent systemic constraints.

**TABLE 1:** Summary of sustainable and disaster-resilient infrastructure *d*imension *s*cores for Ahmad Yani corridor.

Dimension	Score	Interpretation
Risk	0.00	Very low – hazard exposure remains high with minimal integration of disaster risk assessment into infrastructure planning.
Vulnerability	0.43	Low to moderate – partial awareness of critical vulnerabilities, but a lack of systematic prioritisation or mitigation programmes.
Resistance	2.00	Moderate – structural standards are applied, but limited hazard-specific design and maintenance adaptation.
Recovery time	3.29	High – repair and restoration systems are functioning, enabling relatively rapid recovery following disruptions.
Social	3.62	High – infrastructure strongly supports social connectivity, mobility and access, though participation in resilience planning is limited.
Economic	1.50	Low to moderate – reliance on central government funding; limited local financial autonomy or contingency budgeting for resilience.
Environmental	-	Not assessed – road and bridge infrastructure is classified as passive assets with a negligible operational environmental load.

The results show that Bandung’s road and bridge infrastructure demonstrates high performance in reactive capacities (recovery time, social) and moderate technical robustness (resistance), while proactive risk management and vulnerability reduction remain very limited. Economic resilience is weak, and environmental assessment was excluded due to the passive operational nature of the infrastructure type.

### Dimension-level strengths and weaknesses

**FIGURE 3:**
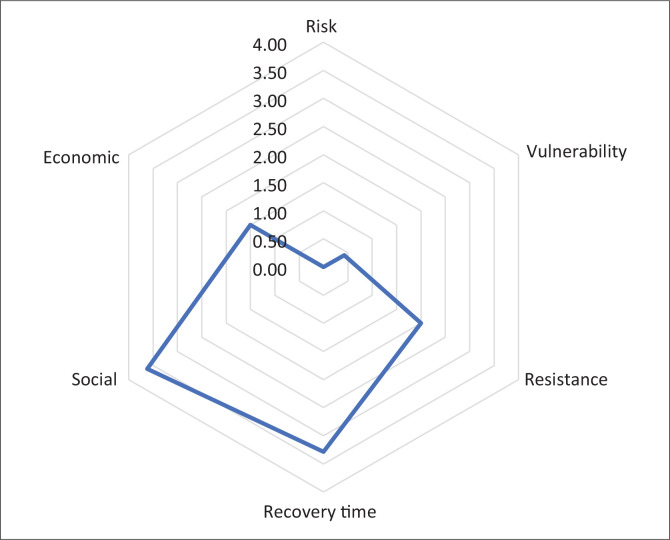
Sustainable and disaster-resilient infrastructure radar diagram.

The indicator-based results reveal substantial variation across the seven SDRI dimensions, underscoring an uneven resilience profile for Bandung’s road and bridge infrastructure (Figure 3). While some dimensions demonstrate operational strength, others expose critical systemic weaknesses that constrain proactive resilience. The following discussion interprets the results of each dimension in relation to local institutional, technical and socio-economic conditions.

### Risk dimension (score 0.00)

The risk dimension records the lowest possible value, signifying a complete absence of structured risk assessment and hazard integration in Bandung’s infrastructure management (Table 2). None of the relevant indicators – ranging from the existence of formal risk assessment procedures to the development of hazard-scenario modelling – is implemented in practice. Although hazard maps produced by the BNPB and the provincial government exist, they are not formally integrated into infrastructure planning or maintenance programmes. Institutional capacity for risk data management and early-warning coordination remains minimal, reflecting fragmented responsibilities among agencies. Consequently, risk management remains reactive rather than anticipatory, leaving the city’s infrastructure exposed to recurring hazards without systematic mitigation or preparedness strategies.

**TABLE 2:** Risk dimension score.

Indicator	Description	Code	Score
Existence and application of disaster risk assessment	Evaluates whether formal disaster risk assessments exist, are regularly updated and integrated into planning, policy and design processes.	[R1]	0.00
Scope of hazard analysis	Assesses the breadth of hazards considered – whether analysis covers single or multiple types of natural and non-natural disturbances.	[R2]	0.00
Organisational capacity for risk data processing and early warning	Measures the institutional ability to manage, analyse and utilise risk data and early-warning systems in decision-making.	[R3]	0.00
Existence and functionality of early-warning systems	Evaluates the presence and operational reliability of early-warning systems for multiple hazards relevant to infrastructure safety.	[R4]	0.00
Development of hazard scenarios	Determines whether hazard-scenario modelling exists and is used to anticipate infrastructure performance under different disaster conditions.	[R5]	0.00

### Vulnerability dimension (score 0.43)

The vulnerability dimension shows only marginal progress beyond the risk category (Table 3). Existing assessments acknowledge exposure to climate and disaster hazards (V1, V2 and V3), yet these insights are not operationalised into adaptive infrastructure measures. Indicators of infrastructure sensitivity (V4) and adaptive capacity (V6) also remain weak, demonstrating limited translation of vulnerability knowledge into design or maintenance standards. Only the absorptive capacity (V5) – which reflects the system’s ability to absorb minor shocks without failure – shows relatively better performance (score 2.00), largely due to the physical robustness of core urban corridors and redundancy in road linkages. However, the absence of formal adaptation programmes or targeted retrofitting underscores a missed opportunity to institutionalise vulnerability reduction. In essence, Bandung recognises its exposure but lacks the institutional mechanisms to convert that awareness into preventive action.

**TABLE 3:** Vulnerability dimension score.

Indicator	Description	Code	Score
Infrastructure vulnerability to climate change impacts	Examines infrastructure’s exposure and susceptibility to climate-related impacts, such as heat, flooding or precipitation changes.	[V1]	0.71
Exposure level to hazards	Measures how exposed infrastructure assets are to potential hazards that could cause service disruption or physical damage.	[V2]	0.00
Infrastructure system risk level	Evaluates how potential damage to one infrastructure component could cascade to affect others within the network.	[V3]	0.00
Infrastructure sensitivity to disaster impacts	Assesses the degree to which infrastructure design, materials or siting increase susceptibility to hazard impacts.	[V4]	0.14
Absorptive capacity of infrastructure	Indicates the infrastructure’s ability to absorb shocks and maintain functionality without significant performance degradation.	[V5] [S4]	2.00
Adaptive capacity of infrastructure	Reflects the ability of infrastructure systems to adjust design, operation or management in response to evolving risks.	[V6]	0.14
Restorative (recovery) capacity of infrastructure	Captures how quickly and effectively infrastructure can return to normal operational levels after disruption.	[V7]	0.00

### Resistance dimension (score 2.00)

The resistance dimension registers a moderate score, reflecting Bandung’s partial success in ensuring structural reliability through compliance with national design standards, including Standar Nasional Indonesia (SNI) [*Indonesian National Standard*] 1725:2016 (bridge loading) and SNI 2833:2016 (seismic resistance) (Table 4). These standards provide a solid baseline for engineering safety under normal conditions (K1, K3). However, hazard-specific reinforcement – especially for bridges in flood-prone or liquefaction-susceptible zones – remains limited. Preventive maintenance practices are largely reactive, conducted only after visible degradation or post-event damage. Consequently, infrastructure performance degradation during disasters remains a recurring concern. The lack of systematic monitoring or retrofitting programmes constrains the long-term durability of structures, leaving physical resistance adequate but far from optimal for multi-hazard resilience.

**TABLE 4:** Resistance dimension score.

Indicator	Description	Code	Score
Performance degradation during disaster events	Measures the extent to which infrastructure maintains functionality and structural integrity during disaster events	[K1] [K3]	2.00

### Recovery time dimension (score 3.29)

Recovery time emerges as one of Bandung’s strongest dimensions, with a score of 3.29 indicating efficient restoration of infrastructure functionality following disruption (Table 5). This outcome is supported by established coordination between the Public Works Department, the Regional Disaster Management Agency and BNPB, which ensures rapid mobilisation for debris clearance, temporary road construction and post-disaster repairs (W1, K2). The integration of maintenance and emergency repair funding in annual regional budgets also reinforces this reactive capability. However, this strength primarily represents response efficiency rather than resilience maturity – in other words, the system recovers quickly but not necessarily more intelligently or sustainably. The absence of post-event learning mechanisms and resilience monitoring limits the opportunity to improve recovery efficiency over time.

**TABLE 5:** Recovery time dimension score.

Indicator	Description	Code	Score
Speed of infrastructure recovery	Evaluates the efficiency and timeliness of post-disaster repair and service restoration processes	[W1] [K2]	3.29

### Social dimension (score 3.62)

The social dimension scores the highest overall (3.62), driven by strong indicators in cultural compatibility and preservation (S1) and community participation and social integration (S3, S6), both scoring 4.00. Bandung’s road and bridge infrastructure plays a crucial social role by facilitating daily mobility, connecting economic zones, and ensuring access to services (Table 6). These networks sustain community interactions and reinforce urban cohesion. However, while infrastructure provides significant social utility, it does not yet guarantee social adaptability. Community engagement in infrastructure planning remains limited to post-disaster recovery and maintenance programmes, rather than to participatory risk reduction. The social strength observed here is thus infrastructural rather than institutional – it reflects the infrastructure’s importance to society, not the society’s involvement in shaping its resilience.

**TABLE 6:** Social dimension score.

Indicator	Description	Code	Score
Cultural compatibility and preservation	Examines how infrastructure projects respect and enhance local cultural values, heritage sites and social identity.	[S1]	4.00
Accessibility and public utility coverage	Assesses the inclusiveness of infrastructure in serving diverse population groups and promoting equitable access.	[S2] [S5]	2.86
Community participation and social integration	Evaluates the degree of community involvement and collaboration in planning, implementation and monitoring.	[S3] [S6]	4.00

### Economic dimension (score 1.50)

The economic dimension underperforms across most indicators, primarily due to the absence of dedicated funding mechanisms for resilience and adaptation (Table 7). The city’s infrastructure financing remains heavily dependent on national transfers and project-based budgeting. Indicators related to reconstruction financing (S13) and economic analysis (S14–S16) show modest readiness, while post-disaster resource projection (S18) scores zero, indicating no established contingency allocation. The budget structure is predominantly output-oriented – prioritising new construction over lifecycle maintenance or resilience investment. This approach creates fiscal vulnerability, leaving limited flexibility for proactive risk-reduction. The absence of resilience-oriented financial planning constrains Bandung’s ability to sustain infrastructure functionality under financial or disaster stress.

**TABLE 7:** Economic dimension score.

Indicator	Description	Code	Score
Preparedness for reconstruction financing	Evaluates financial readiness and adequacy of reconstruction funds, insurance or contingency mechanisms.	[S13]	2.00
Comprehensiveness of economic analysis	Determines whether the infrastructure economic evaluation considers multiple aspects such as cost, benefit and sustainability.	[S14] [S15] [S16]	2.00
Additional technical benefits	Identifies supplementary technical gains from infrastructure, such as innovation, capacity building or co-benefits.	[S17]	2.00
Projection of post-disaster resource availability	Assesses the adequacy and continuity of material and financial resources available for reconstruction after disasters.	[S18]	0.00

### Environmental dimension (excluded)

The environmental dimension was excluded from quantitative scoring due to the passive operational nature of road and bridge infrastructure, which consumes minimal resources during use. Nonetheless, environmental considerations – such as material sustainability, runoff management and urban heat mitigation – remain indirectly relevant. Future cross-sectoral applications should reincorporate this dimension when assessing hybrid or green–grey infrastructure systems to capture environmental–technical interdependencies.

### Synthesis and interpretation

Overall, the dimension-level analysis confirms that Bandung’s infrastructure resilience is reactive and partial. High scores in social and recovery time dimensions illustrate strong operational and community functionality, while near-zero values in risk and vulnerability expose systemic weaknesses in anticipatory governance and prevention. Moderate resistance and weak economic performance further highlight the absence of sustained investment in long-term durability. This pattern reflects a city capable of bouncing back but not yet able to adapt to the future. Enhancing resilience, therefore, requires institutionalising risk-based planning, diversifying funding streams and embedding participatory mechanisms in infrastructure governance to transition from reactive recovery towards adaptive sustainability.

### Interrelationship between sustainability and resilience dimensions

The inter-dimensional pattern reveals a reactive-over-proactive imbalance in Bandung’s infrastructure resilience.

The highest scores in recovery time (3.29) and social (3.62) stand in sharp contrast to the near-zero values for risk and vulnerability, confirming that the city’s infrastructure system prioritises restoration after damage over risk avoidance before impact. This pattern is typical in cities where institutional focus and funding mechanisms are oriented towards emergency response rather than prevention (Gillespie-Marthaler et al. 2019; Nelson et al. 2020).

The resistance dimension functions as the intermediate link between proactive and reactive capacities. While structural robustness enables the infrastructure to withstand moderate disturbances, the lack of preventive retrofitting and hazard-specific upgrades limits its ability to reduce long-term vulnerability. The Economic dimension’s weakness (1.50) compounds this issue – without predictable local funding sources, preventive resilience investments are rare.

Interestingly, the social dimension’s high score highlights a paradox: socially beneficial infrastructure (in terms of accessibility and connectivity) may still be resilience-deficient without support from risk governance, community preparedness or economic continuity mechanisms. This emphasises the need to integrate social utility with social adaptability – ensuring that infrastructure not only connects people but also protects them.

Overall, the interplay between dimensions suggests that Bandung’s road and bridge system exhibits reactive strength but anticipatory weakness. The city’s ability to recover quickly conceals systemic exposure to recurring risk and financial fragility. This imbalance highlights the need to transition from a ‘build–repair’ model to a ‘plan–anticipate–adapt’ model of infrastructure resilience.

### Summary of findings

The pilot application of the SDRI framework reveals that Bandung’s road and bridge infrastructure exhibits a highly unbalanced resilience profile. The city performs strongly in reactive dimensions, particularly in recovery time and social aspects, which demonstrate improved emergency responsiveness, effective repair mechanisms and the vital social function of maintaining connectivity and mobility during disruptions. Structural robustness (Resistance) is moderate, as infrastructure generally adheres to national design standards such as SNI 1725:2016 and SNI 2833:2016, but hazard-specific adaptation remains limited. In contrast, proactive dimensions, namely risk and vulnerability, score poorly, reflecting the absence of comprehensive hazard-mapping integration and preventive risk-reduction strategies in infrastructure planning. Socio-economic enablers, particularly economic resilience, remain weak due to the lack of dedicated funding for disaster preparedness and limited community involvement in resilience-oriented decision-making. Environmental considerations are excluded from the analysis because road and bridge infrastructure is classified as a passive asset that does not require operational resource inputs, although these aspects should be considered in future multi-sectoral assessments.

Overall, Bandung’s infrastructure resilience can be characterised as reactive and partial – technically capable of recovery but lacking anticipatory capacity and systemic integration. This imbalance reflects a common pattern among developing cities, where institutional and fiscal constraints lead to a focus on post-disaster recovery rather than proactive prevention. The findings demonstrate that the SDRI framework can identify multi-dimensional gaps and interdependencies, even under data-limited conditions. It provides actionable insights for policymakers and practitioners: enhancing Bandung’s resilience requires mainstreaming risk-based planning into infrastructure design and budgeting, fostering broader community participation and public awareness, and establishing dedicated financial mechanisms for preventive adaptation. By clarifying where resilience is strong and where it fails, this pilot study validates the SDRI framework’s diagnostic value and lays the groundwork for future, more comprehensive stakeholder-based assessments across multiple urban contexts.

## Discussion

### Interpreting Bandung’s sustainable and disaster-resilient infrastructure performance

The Bandung pilot application reveals an asymmetric resilience profile dominated by reactive strength rather than proactive preparedness. The highest-performing dimensions – recovery time (3.29) and social (3.62) –reflect the city’s strong capacity to restore infrastructure functionality and maintain essential connectivity after disruptions. This is largely supported by established coordination between municipal and national disaster response agencies, which enables rapid recovery and service continuity. However, these strengths stand in sharp contrast to the lowest scores for risk (0.00) and vulnerability (0.43), indicating a lack of systematic hazard-based planning, limited analysis of asset exposure and minimal preventive adaptation. The resistance score (2.00) indicates moderate compliance with national design standards, but without explicit adjustments for site-specific hazards such as earthquakes or floods. Meanwhile, economic resilience (1.50) remains constrained by dependence on central funding, revealing structural fiscal vulnerability.

This configuration suggests that Bandung’s infrastructure resilience is reactive and operational, oriented toward restoration rather than anticipation. The city’s roads and bridges perform well under normal conditions and can recover relatively quickly after disruptions, but they remain exposed to recurrent risks. The results confirm that Bandung’s resilience model prioritises response efficiency over risk reduction, reflecting a broader developmental pattern in many Indonesian and other developing cities, where institutional and financial constraints limit the implementation of proactive resilience measures.

### Comparison with existing studies

The pattern observed in Bandung aligns closely with global research on urban resilience in developing contexts. Bocchini et al. (2014) identified a consistent tendency for infrastructure systems in resource-constrained environments to focus on bouncing back rather than building forward. The dominance of reactive dimensions, like recovery time, demonstrates what Cutter, Ash and Emrich (2014) term a ‘resilience maturity gap’ – cities tend to develop technical and operational capacities before institutionalising proactive governance and risk financing mechanisms.

The relatively strong resistance and social scores further confirm observations by Ahern (2011) and Meerow, Newell and Stults (2016) that early-stage resilience in developing countries is often achieved through engineering robustness and social utility rather than through integrated governance or financial resilience. However, the persistence of weak risk and vulnerability dimensions in Bandung suggests that resilience remains situational rather than systemic. Without embedding risk information and vulnerability mapping into planning, infrastructure resilience will remain reactive to crises rather than adaptive to evolving threats.

This finding is consistent with previous studies in another big Indonesian city, such as Puspita and Pamungkas (2025) assessment of institutional resilience in Surabaya, which demonstrates that the city prioritises efficient emergency response systems – such as coordinated disaster response units and 24/7 command centres – over the systematic integration of disaster risk reduction into long-term urban planning. This pattern reflects a broader trend in Indonesian cities, where fiscal limitations and institutional constraints hinder the shift from reactive responses to anticipatory, risk-informed infrastructure planning.

This finding also complements national-level assessments, such as those by Trigunarsyah (2021), which highlights Indonesia’s gap between disaster management regulations and their operationalisation at the municipal level. Bandung exemplifies this disconnect: national frameworks (e.g. BNPB disaster protocols, SNI standards) exist, but local implementation and data integration remain limited.

Recent governance analyses further reinforce this pattern by showing that disaster resilience in Indonesia is predominantly operationalised through response and recovery mechanisms rather than structural risk reduction. This reflects systemic issues, including fragmented institutional arrangements and limited integration of risk data into planning processes, which constrain the shift toward anticipatory resilience (Setyawati 2024).

Similarly, broader development analyses indicate that local governments in Indonesia tend to prioritise short-term, visible interventions – such as emergency response improvements – over long-term risk reduction. This is driven by limited technical capacity, fragmented institutional mandates and constrained municipal budgets, all of which restrict the scope for proactive and preventive resilience planning (Kryspin-Watson et al. 2019).

### Practical implications for infrastructure planning

The results have direct implications for infrastructure planning and governance. Firstly, the consistently low risk and vulnerability scores underline the urgent need to mainstream risk-informed planning into the municipal infrastructure development process. Integrating hazard and exposure data into project design, budgeting, and prioritisation would allow Bandung to transition from a reactive maintenance model to a proactive risk-reduction regime.

Secondly, the weak economic dimension calls for dedicated resilience financing mechanisms, such as contingency funds, performance-based maintenance reserves or resilience bonds. Without predictable local funding streams, preventive adaptation will remain sidelined by more immediate construction demands. Strengthening economic resilience, therefore, requires fiscal innovation and regulatory reform.

Thirdly, the high social score points to a significant opportunity: Bandung’s infrastructure already supports strong social connectivity and mobility. This can be leveraged to create a more adaptive governance model, where public participation, community awareness and transparency are embedded in infrastructure decision-making. By institutionalising participatory processes, the city can enhance both social legitimacy and collective preparedness.

In this way, Bandung’s case serves as a bridge to the broader governance discussion that will be elaborated in subsequent research. It demonstrates that resilience must evolve from an engineering-based concept to a governance-based practice – linking technical infrastructure performance with institutional capacity, social inclusion and financial accountability.

### Methodological reflections

Methodologically, the Bandung pilot underscores the operational value of the SDRI framework in data-limited contexts. Despite relying solely on secondary data and expert judgement, the framework effectively identified cross-dimensional imbalances and actionable priorities. This validates its design principle from Firdaus et al. (2025): that an integrated sustainability–resilience assessment can remain functional even when quantitative data are sparse, provided indicators are clearly defined and contextually interpretable.

The exclusion of the environmental dimension for passive infrastructure (roads and bridges) further demonstrates the framework’s contextual flexibility. By allowing indicator selection to reflect infrastructure characteristics, the SDRI framework avoids over-generalisation and improves relevance. However, this approach also emphasises the need for transparent justification for the exclusion of indicators to maintain analytical credibility.

The reliance on a single-assessor judgement approach, while pragmatic for a pilot study, introduces potential subjectivity. Future applications should include multi-stakeholder validation or Delphi-based consensus scoring to improve reliability. Additionally, integrating spatial datasets and time-series maintenance records would strengthen the quantitative rigour of future assessments.

### Future research

This study establishes a foundation for several future research directions. Firstly, expanding the SDRI framework’s application to other infrastructure sectors – such as water supply, energy or public transport – would test its generalizability and cross-sector adaptability. Secondly, conducting comparative analyses across multiple cities in Indonesia could reveal structural patterns and contextual determinants of resilience performance, supporting national policy harmonisation. Thirdly, subsequent studies should explore the governance dimensions underlying resilience outcomes – how institutional coordination, leadership and community participation influence the translation of resilience principles into practice.

Finally, integrating the SDRI framework with spatial and probabilistic risk modelling could advance its diagnostic precision. By coupling qualitative assessment with quantitative simulations of hazard impacts and recovery trajectories, future research can evolve from descriptive profiling toward predictive resilience analytics. This would position the SDRI framework as both a decision-support and policy-monitoring tool, linking empirical infrastructure assessment with adaptive governance strategies.

### Limitations

This study, while providing valuable insights into the practical application of the SDRI framework, is subject to several limitations that should be acknowledged. Firstly, the assessment relied primarily on secondary data and the author’s expert judgement, without multi-stakeholder validation. Although this approach was appropriate for a pilot application, it introduces the potential for subjective interpretation, particularly in scoring qualitative indicators such as vulnerability, social participation and economic resilience. Future research should employ participatory evaluation methods – such as Delphi techniques or focus group discussions – to enhance inter-rater reliability and minimise bias.

Secondly, the limited availability and consistency of local infrastructure data constrained the scope of indicator quantification. Several indicators were evaluated qualitatively due to gaps in long-term performance records or spatially disaggregated datasets. This limitation reflects a broader challenge in developing-country contexts, where infrastructure and risk data are fragmented across multiple institutions. While the framework demonstrated robustness in handling incomplete data, further validation with more comprehensive and standardised datasets would strengthen its analytical accuracy.

Thirdly, the exclusion of the Environmental dimension from this assessment, though justified by the passive operational nature of road and bridge infrastructure, reduces the holistic completeness of the SDRI model. Environmental interactions – particularly those related to construction materials, land-use change and stormwater management – could indirectly influence resilience outcomes. Future applications should therefore integrate these interactions when assessing multi-sectoral infrastructure systems or hybrid green–grey solutions.

Fourthly, the single-case study design limits the generalizability of findings. Bandung’s urban, institutional and fiscal context may not fully represent other Indonesian cities, especially smaller municipalities or those with differing hazard exposures. Nonetheless, as a pilot application, the case provides a replicable methodological template that can be adapted for broader comparative studies across varying spatial and governance settings.

Finally, the SDRI framework’s static assessment structure – based on current conditions – does not yet capture temporal evolution or dynamic interdependencies between dimensions. Infrastructure resilience is inherently time-dependent, influenced by changing hazard profiles, policy shifts and socio-economic transitions. Incorporating temporal analysis and scenario-based modelling in future research would enhance the framework’s ability to evaluate not only current resilience but also its trajectory over time.

Despite these limitations, the study successfully demonstrates the SDRI framework’s operational feasibility and diagnostic capacity in a real-world urban setting with data constraints. The identified limitations do not undermine the validity of the findings; rather, they highlight essential directions for advancing methodological rigour, multi-actor engagement and dynamic modelling in subsequent research phases.

## Conclusion

This study applied the SDRI framework to evaluate the resilience performance of road and bridge infrastructure in Bandung City, Indonesia. As one of the first empirical applications of the framework, it demonstrated how multi-dimensional resilience assessment can be operationalised using secondary data and structured expert judgement in a developing-country context. The findings reveal a distinctly reactive resilience profile – marked by strong recovery and social functions but weak proactive capacities in risk management, vulnerability reduction and financial preparedness.

The analysis showed that Bandung’s infrastructure performs well in recovery time and social dimensions, indicating efficient post-disaster repair systems and strong social connectivity supported by transportation networks. The resistance dimension scored moderately, reflecting adherence to technical standards but limited adaptation of hazard-specific design. In contrast, the lowest scores in risk, vulnerability and economic dimensions highlight the absence of integrated hazard-based planning, proactive mitigation and sustainable funding mechanisms. The environmental dimension was not assessed due to the passive operational nature of the infrastructure type, though future multi-sectoral studies should reintroduce it to ensure a comprehensive sustainability–resilience linkage.

Theoretically, this study reinforces the importance of viewing infrastructure resilience as a multi-dimensional and dynamic construct that extends beyond structural robustness to encompass social, institutional and economic capacities. The Bandung case illustrates how resilience can exist in a reactive equilibrium, where systems recover quickly yet remain persistently exposed to recurring risks. This supports the broader argument that urban infrastructure resilience cannot be achieved through engineering interventions alone – it requires anticipatory governance, financial foresight and participatory engagement.

Methodologically, the study validates the practical usability of the SDRI framework for data-constrained environments. Its structured, indicator-based design enabled transparent, reproducible evaluation, even with incomplete datasets. The framework’s flexibility to exclude non-applicable dimensions (such as Environmental in this case) demonstrates its adaptability across infrastructure types and contexts. However, a key limitation of this study is its reliance on the authors’ expert judgement in certain stages of the assessment, which may introduce subjectivity into scoring. Future research should incorporate multi-stakeholder validation processes, such as expert workshops or Delphi studies, to refine and validate the assessment parameters and scoring mechanisms. Such approaches would enhance the robustness, transparency and contextual relevance of the framework, particularly in diverse institutional settings, particularly in decentralised governance contexts where multiple stakeholders influence infrastructure decision-making.

From a policy perspective, the findings underscore three urgent priorities for improving infrastructure resilience in Bandung and similar cities:

- Integrate risk and vulnerability mapping into early stages of infrastructure planning and investment decision-making.
- Develop dedicated resilience financing mechanisms at the municipal level to reduce dependence on reactive post-disaster funding.
- Institutionalise participatory and transparent governance in infrastructure management to strengthen social and institutional adaptive capacity.

In conclusion, this study demonstrates that the SDRI framework is not only conceptually comprehensive but also practically actionable. It enables policymakers and practitioners to identify where resilience is strong, where it is deficient, and how it can be strategically improved. The Bandung pilot provides a replicable methodological model for cities seeking to operationalise sustainability and resilience principles within their infrastructure systems – transforming resilience from a reactive aspiration into an embedded planning practice.

## References

[CIT0001] Ahern, J., 2011, ‘From fail-safe to safe-to-fail: Sustainability and resilience in the new urban world’, *Landscape and Urban Planning* 100(4), 341–343. 10.1016/j.landurbplan.2011.02.021

[CIT0002] Bandung, B.K., 2020, *Laporan Evaluasi Kinerja Infrastruktur Kota Bandung Tahun 2020* [Bandung City infrastructure performance evaluation report 2020], Pemerintah Kota Bandung, Bandung.

[CIT0003] Bocchini, P., Frangopol, D.M., Ummenhofer, T. & Zinke, T., 2014, ‘Resilience and sustainability of civil infrastructure: Toward a unified approach’, *Journal of Infrastructure Systems* 20(2), 04014004. 10.1061/(ASCE)IS.1943-555X.0000177

[CIT0004] Bruneau, M., Chang, S.E., Eguchi, R.T., Lee, G.C., O’Rourke, T.D., Reinhorn, A.M. et al., 2003, ‘A framework to quantitatively assess and enhance the seismic resilience of communities’, *Earthquake Spectra* 19, 733–752. 10.1193/1.1623497

[CIT0005] Chhibber, A. & Laajaj, R., 2007, *Natural disasters and economic development impact, response and preparedness*, Global Development Network, viewed n.d., from https://www.researchgate.net/profile/Rachid-Laajaj/publication/265198864_Natural_Disasters_and_Economic_Development_Impact_Response_and_Preparedness/links/54d8d7cb0cf2970e4e79d610/Natural-Disasters-and-Economic-Development-Impact-Response-and-Preparedness.pdf.

[CIT0006] Cutter, S.L., Ash, K.D. & Emrich, C.T., 2014, ‘The geographies of community disaster resilience’, *Global Environmental Change* 29, 65–77. 10.1016/j.gloenvcha.2014.08.005

[CIT0007] Firdaus, A., Pribadi, K.S., Abduh, M. & Sagala, S., 2025, ‘Developing a sustainable and disaster resilient infrastructure assessment framework’, *International Postgraduate Conference on Civil Engineering Proceedings* 1(1), 30–37, viewed n.d., from https://ipcce.ftsl.itb.ac.id/index.php/proceedings/issue/view/home.

[CIT0008] Gibson, R.B., 2006, ‘Beyond the pillars: Sustainability assessment as a framework for effective integration of social, economic and ecological considerations in significant decision-making’, *Journal of Environmental Assessment Policy and Management* 8(3), 259–280. 10.1142/S1464333206002517

[CIT0009] Gillespie-Marthaler, L., Nelson, K.S., Baroud, H., Kosson, D.S. & Abkowitz, M., 2019, ‘An integrative approach to conceptualizing sustainable resilience’, *Sustainable and Resilient Infrastructure* 4(2), 66–81. 10.1080/23789689.2018.1497880

[CIT0010] Google Maps, 2025, *Jalan Ahmad Yani, Bandung* [Ahmad Yani Street, Bandung], Google, viewed 10 September 2025, from https://www.google.com/maps/place/Jl.+A.+Yani,+Kota+Bandung,+Jawa+Barat/@-6.9117864,107.6360984,3468m/data=!3m1!1e3!4m10!1m2!2m1!1sjalan+ahmad+yani+bandung!3m6!1s0x2e68e7c3dac3b8a1:0x54d5e2aabe5d5446!8m2!3d-6.9112326!4d107.6384802!15sChhqYWxhbiBhaG1hZCB5YW5pIGJhbmR1bmeSAQVyb3V0ZeABAA!16s%2Fg%2F1hm4vl8l8?entry=ttu&g_ep=EgoyMDI2MDMxNy4wIKXMDSoASAFQAw%3D%3D.

[CIT0011] Holling, C.S., 1973, ‘Resilience and stability of ecological systems’, *Annual Review of Ecology and Systematics* 4, 1–23. 10.1146/annurev.es.04.110173.000245

[CIT0012] Husnita, N. & Ghaniyyu, F.F., 2020, ‘Implementasi Konsep Pembangunan Berkelanjutan Terhadap Penguatan Konsep Dasar Hukum Penataan Ruang Berdasarkan Fungsi Lingkungan Hidup’ [The implementation of sustainable development concepts to strengthen the fundamental legal framework for spatial planning based on environmental functions], *Padjadjaran Law Review* 8, 20–39.

[CIT0013] Khazai, B., Anhorn, J. & Burton, C.G., 2018, ‘Resilience Performance Scorecard: Measuring urban disaster resilience at multiple levels of geography with case study application to Lalitpur, Nepal’, *International Journal of Disaster Risk Reduction* 31, 604–616. 10.1016/j.ijdrr.2018.06.012

[CIT0014] Kryspin-Watson, J., Vun, Y.J., Stanton-Geddes, Z. & Semadeni, G.S., 2019, *Strengthening the disaster resilience of Indonesian cities: A policy note*, World Bank Group, Washington, DC, viewed n.d., from http://documents.worldbank.org/curated/en/748581569515561529.

[CIT0015] Lee, S.H., An, L.-S. & Kim, H.-K., 2025, ‘Risk-based bridge life cycle cost and environmental impact assessment considering climate change effects’, *Scientific Reports* 15, 725. 10.1038/s41598-024-82568-4 39753640 PMC11698832

[CIT0016] Meerow, S., Newell, J.P. & Stults, M., 2016, ‘Defining urban resilience: A review’, *Landscape and Urban Planning* 147, 38–49. 10.1016/j.landurbplan.2015.11.011

[CIT0017] National Disaster Management Agency (BNPB), 2021, *Bencana Indonesia 2016–2020* [Disasters in Indonesia, 2016–2020], viewed 06 August 2025, from https://www.bnpb.go.id/infografis.

[CIT0018] Nelson, K., Gillespie-Marthaler, L., Baroud, H., Abkowitz, M. & Kosson, D., 2020, ‘An integrated and dynamic framework for assessing sustainable resilience in complex adaptive systems’, *Sustainable and Resilient Infrastructure* 5(5), 311–329. 10.1080/23789689.2019.1578165

[CIT0019] Puspita, F.S.P. & Pamungkas, A., 2025, ‘Assessing institutional resilience index of Surabaya City against earthquake risk: A case study using the CDRI framework’, *Jurnal Penataan Ruang* 20, 130–142. 10.12962/j2716179X.v20iI.5504

[CIT0020] Setyawati, L.A., 2024, ‘Strengthening disaster resilience in Indonesia: A framework for sustainable recovery through the pentahelix model’, *Information, Communications, and Disaster* 1(2), 85–93. 10.61511/icd.v1i2.2024.1944

[CIT0021] Shrestha, B.B. & Kawasaki, A., 2020, ‘Quantitative assessment of flood risk with evaluation of the effectiveness of dam operation for flood control: A case of the Bago River Basin of Myanmar’, *International Journal of Disaster Risk Reduction* 50, 101707. 10.1016/j.ijdrr.2020.101707

[CIT0022] Statistik, B.P., 2022, *Kota Bandung dalam Angka 2022* [Bandung City in figures 2022], BPS Kota Bandung, Bandung.

[CIT0023] Trigunarsyah, B., 2021, ‘Hambatan Penerapan Konstruksi Berkelanjutan: Perspektif Pemerintah’ [Barriers to the implementation of sustainable construction: A government perspective], *Media Komunikasi Teknik Sipil* 27(1), 18–28. 10.14710/mkts.v27i1.33764

[CIT0024] United Nations Development Programme, 2015, *Sendai framework for disaster risk reduction 2015–2030*, UNDRR, Geneva.

[CIT0025] United Nations Environment Programme, 2021, *International good practice principles for sustainable infrastructure*, viewed 16 July 2025, from https://www.unep.org/resources/publication/international-good-practice-principles-sustainable-infrastructure.

[CIT0026] Vallery, H., 2020, *Implementasi sustainable development goals (SDGs) di Indonesia selama pemerintahan Presiden Joko Widodo periode 2015–2019* [Implementation of sustainable development goals (SDGs) in Indonesia under President Joko Widodo period 2015-2019], viewed n.d., from https://repository.uph.edu.

[CIT0027] World Commission on Environment and Development, 1987, ‘World commission on environment and development’, *Our Common Future* 17(1), 1–91.

